# Advances in cancer DNA methylation analysis with methPLIER: use of non-negative matrix factorization and knowledge-based constraints to enhance biological interpretability

**DOI:** 10.1038/s12276-024-01173-7

**Published:** 2024-03-04

**Authors:** Ken Takasawa, Ken Asada, Syuzo Kaneko, Kouya Shiraishi, Hidenori Machino, Satoshi Takahashi, Norio Shinkai, Nobuji Kouno, Kazuma Kobayashi, Masaaki Komatsu, Takaaki Mizuno, Yu Okubo, Masami Mukai, Tatsuya Yoshida, Yukihiro Yoshida, Hidehito Horinouchi, Shun-Ichi Watanabe, Yuichiro Ohe, Yasushi Yatabe, Takashi Kohno, Ryuji Hamamoto

**Affiliations:** 1 Division of Medical AI Research and Development, National Cancer Center Research Institute, Tokyo, 104-0045 Japan; 2 Cancer Translational Research Team, RIKEN Center for Advanced Intelligence Project, Tokyo, 103-0027 Japan; 3 Division of Genome Biology, National Cancer Center Research Institute, Tokyo, 104-0045 Japan; 4 Department of Thoracic Oncology, National Cancer Center Hospital, Tokyo, 104-0045 Japan; 5 Department of Experimental Therapeutics, National Cancer Center Hospital, Tokyo, 104-0045 Japan; 6 Department of Thoracic Surgery, National Cancer Center Hospital, Tokyo, 104-0045 Japan; 7 Division of Medical Informatics, National Cancer Center Hospital, Tokyo, 104-0045 Japan; 8 Department of Diagnostic Pathology, National Cancer Center Hospital, Tokyo, 104-0045 Japan

**Keywords:** Computational platforms and environments, Non-small-cell lung cancer

## Abstract

DNA methylation is an epigenetic modification that results in dynamic changes during ontogenesis and cell differentiation. DNA methylation patterns regulate gene expression and have been widely researched. While tools for DNA methylation analysis have been developed, most of them have focused on intergroup comparative analysis within a dataset; therefore, it is difficult to conduct cross-dataset studies, such as rare disease studies or cross-institutional studies. This study describes a novel method for DNA methylation analysis, namely, methPLIER, which enables interdataset comparative analyses. methPLIER combines Pathway Level Information Extractor (PLIER), which is a non-negative matrix factorization (NMF) method, with regularization by a knowledge matrix and transfer learning. methPLIER can be used to perform intersample and interdataset comparative analysis based on latent feature matrices, which are obtained via matrix factorization of large-scale data, and factor-loading matrices, which are obtained through matrix factorization of the data to be analyzed. We used methPLIER to analyze a lung cancer dataset and confirmed that the data decomposition reflected sample characteristics for recurrence-free survival. Moreover, methPLIER can analyze data obtained via different preprocessing methods, thereby reducing distributional bias among datasets due to preprocessing. Furthermore, methPLIER can be employed for comparative analyses of methylation data obtained from different platforms, thereby reducing bias in data distribution due to platform differences. methPLIER is expected to facilitate cross-sectional DNA methylation data analysis and enhance DNA methylation data resources.

## Breaking Bias in DNA Methylation Data: The methPLIER Method

DNA methylation is significant in diseases like cancer. However, its full impact on gene activity is not completely understood. This research, led by Ken Takasawa and Ryuji Hamamoto, aimed to create a new method to examine DNA methylation data for better comprehension of its biological relevance. The team utilized a technique named "methPLIER," an analysis type that compares various DNA methylation data sets. They examined data from 9,756 cancer samples, focusing on how DNA methylation near gene beginnings might regulate gene activity. The scientists used a unique approach to condense the data and then applied methPLIER to discover patterns that could clarify biological processes. They concluded that this technique could aid in understanding the biological significance of DNA methylation patterns. Looking forward, methPLIER could be crucial for future disease research and might assist in developing new cancer diagnosis and treatment methods.

This summary was initially drafted using artificial intelligence, then revised and fact-checked by the author.

## Introduction

DNA methylation – i.e., the addition of a methyl group to the 5’ end of cytosine at CpG sites – is a type of epigenetic modification conserved across prokaryotes and eukaryotes^1^. In eukaryotes, DNA hypermethylation near transcription start sites (TSSs) and around promoters has been reported to suppress gene expression by preventing the binding of transcription factors and other transcriptional regulators, thus contributing to the formation of cell- and tissue-specific gene expression patterns during embryogenesis and cell differentiation^2^. The relationship between gene expression and DNA methylation patterns has also been extensively explored in the cancer research field^3^. Comprehensive DNA methylation analysis methods include methylated DNA immunoprecipitation (MeDIP), the Illumina HumanMethylation BeadChip, reduced-representation bisulfite sequencing (RRBS), and whole-genome bisulfite sequencing (WGBS)^4,5^. The methylated DNA data acquired on these platforms are registered in the Gene Expression Omnibus (GEO) and Sequence Read Archive (SRA), with Illumina HumanMethylation BeadChip analysis data being the most commonly registered and published. As of April 15, 2023, the HumanMethylation450 BeadChip (HM450) has been used to generate 114,799 samples registered in the GEO database, making it a widely used tool in the fields of developmental biology and cancer research.

In HM450 data analysis, raw data are generally normalized by preprocessing, followed by comparative and clustering analyses. Several preprocessing methods have been proposed to date, with different researchers and projects adopting different preprocessing methods^6–8^. Therefore, when performing DNA methylation analysis using public data, it is necessary to preprocess the raw data first. However, preprocessing DNA methylation data from hundreds to thousands of samples requires considerable computational power, thus making cross-dataset analysis difficult. In addition, some datasets registered in GEO are available in the form of raw data, while others are available only in the form of preprocessed data, thus requiring comparative analysis between data processed via different preprocessing methods. Importantly, HM450 data have been reported to have different data distributions depending on the preprocessing method used^9–11^, which may compromise the reproducibility and scientific reproducibility of dataset comparison studies and meta-analyses using these data. The problem of preprocessing-derived bias in data distribution has been established not only for DNA methylation data but also for transcriptomics data. For example, the platform recount2 (https://jhubiostatistics.shinyapps.io/recount/)^12^, which provides transcriptome data processed through a unified pipeline, and MultiPLIER^13^, an analysis method based on latent features generated from large datasets, were developed to perform comparative analysis between datasets with data distribution bias. MultiPLIER first obtains a latent matrix via non-negative matrix factorization (NMF)^14^ of large gene expression data obtained from recount2 with regularization to sparsely include knowledge matrices from gene sets for various pathways and ontologies. Then, through matrix decomposition of the dataset to be analyzed using a latent matrix, datasets from different domains are represented by the same latent feature vector to achieve interdataset comparative analysis.

**Fig. 1:**
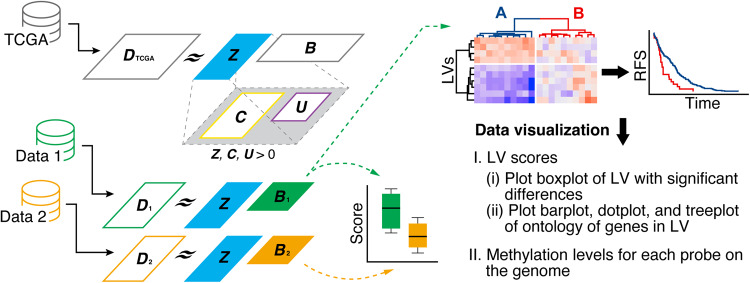
Workflow of our proposed DNA methylation analysis method called “methPLIER”. The DNA methylation data of 9756 samples obtained from TCGA are matrix decomposed via non-negative matrix factorization under the condition that the knowledge matrix, ***C***, is regularized to sparsely include the DNA methylation data and that the latent matrix, ***Z***, is obtained. The obtained ***Z*** is used to matrix decompose the analysis dataset to obtain the loading matrix, which is used for inter- and intradataset comparisons.

In this study, we applied the MultiPLIER architecture to DNA methylation data to develop a novel DNA methylation analysis architecture with high biological semantic interpretability, thus enabling easy interdataset comparative analysis. We named this analysis architecture “methPLIER” and evaluated its data analysis capacity and biological interpretability. methPLIER is a method for performing NMF under regularization conditions that sparsely includes knowledge matrices composed of gene sets such as pathways and ontologies. Since multiple analysis probes are designed for a single gene or transcript in HM450 data, it is necessary to compress the data from probe-wise data to gene-wise data for regularized NMF with a knowledge matrix. Since the analysis of DNA methylation patterns near the transcription start sites of transcripts is essential for DNA methylation analysis, we compressed the data into principal component scores for the analysis probes located 1500 bps from the TSSs, transforming them into gene-wise data for each transcript. In addition, mapping DNA methylation probe data near significant genes onto the genome made it possible to observe the spatial relationship between DNA methylation and expression patterns (Fig. 1). Using methPLIER, we analyzed a lung cancer dataset obtained from the GEO and obtained latent variables (LVs) related to relapse-free survival (RFS). The gene sets included in the dataset were easy to interpret biologically. Furthermore, comparative analysis between different preprocessing methods and different platforms using methPLIER suggested that distributional differences between the datasets could be reduced. Based on the above findings, we believe that DNA methylation analysis using methPLIER is useful for interdataset comparisons and for understanding the biological significance of DNA methylation based on available data.

## Materials and methods

### Dataset used in this study

For the development of methPLIER, DNA methylation data from 9756 samples of different cancers (lung squamous cell carcinoma; lung adenocarcinoma; ovarian cancer; uterine corpus endometrial carcinoma; glioblastoma multiforme; kidney renal clear cell carcinoma; invasive breast carcinoma; thyroid cancer; low-grade glioma; skin cutaneous melanoma; kidney renal papillary cell carcinoma; cervical squamous cell carcinoma and endocervical adenocarcinoma; liver hepatocellular carcinoma; sarcoma; kidney chromophobe; acute myeloid leukemia; adrenocortical carcinoma; mesothelioma; lymphoid neoplasm diffuse large B-cell lymphoma; esophageal carcinoma; head and neck squamous cell carcinoma; prostate adenocarcinoma; colon adenocarcinoma; pheochromocytoma and paraganglioma; uterine carcinosarcoma; uveal melanoma; rectum adenocarcinoma; cholangiocarcinoma; testicular germ cell tumors; thymoma; pancreatic adenocarcinoma; bladder urothelial carcinoma; stomach adenocarcinoma) obtained from The Cancer Genome Atlas (TCGA; and https://portal.gdc.gov) were used. To demonstrate the use of methPLIER, we used a LUAD dataset (GSE39279)^15^ obtained from the Gene Expression Omnibus (GEO; https://www.ncbi.nlm.nih.gov/geo/)^16^. For preprocessing and comparative analysis, we used a clear cell renal cell carcinoma dataset (GSE61441)^17^ obtained from the GEO. For platform comparative analysis, we used HM450, WGBS, and RRBS data from 4 cell lines—GM12878, H1-hESC, HepG2, and SK-N-SH—obtained from the Encyclopedia of DNA Elements (ENCODE; https://www.encodeproject.org)^18^. The Public/Private R&D Investment Strategic Expansion PrograM (PRISM) database is one of the largest lung cancer databases in the world (in-house database of NCC Japan) and contains clinical information from 1714 lung cancer patients, whole-exome analysis data from 1559 lung cancer patients, and RNA-seq data from 1682 lung cancer patients. In addition to clinical information, total exome analysis, and RNA-seq analysis, the PRISM database features whole-genome analysis data for 413 patients, DNA methylation analysis data for 402 patients, and ChIP-seq analysis (H3K27Ac) data for 222 patients (as of April 15, 2023).

### Comparison of DNA methylation data

The HM450 data are expressed as numerical data of DNA methylation status from 0 to 1 for 485,577 probes. To apply the regularization condition via the knowledge matrix, the data were compressed from probe-by-probe data to promoter methylation pattern data for each transcript. Data compression was performed through the following two steps. First, DNA methylation data for analysis probes located within 1500 bps of the transcription start site (TSS) for each transcript were extracted. These analysis probes were subsequently used to perform principal component analysis (PCA) to compress the obtained principal component scores (PCSs) down to the point where the cumulative contribution rate exceeded 80%. The eigenvectors for each principal component axis were used for data compression of the new data. In the feasibility study for compressing gene-wise data, probes located in the 1st exon were used. The compression of gene-wise data from probewise data was conducted using the previously described method.

### Curation of knowledge matrices

The knowledge matrix used for methPLIER consisted of 817 gene sets obtained from the Molecular Signatures Database (MSigDB, https://www.gsea-msigdb.org/gsea/msigdb/)^19–21^. For the knowledge matrix, we used four integrated datasets, namely, bloodCellMarkersIRISDMAP, svmMarkers, canonicalPathways, and oncogenicPathways, which were incorporated into the R package PLIER^22^. The knowledge matrix included 545 biological pathways^23^, 83 cell- and tissue-specific genetic markers^24^, 189 cancer-related pathways^25^, and 817 gene sets. The methPLIER-CTD was constructed using a gene set for 2545 adenocarcinoma-related chemicals obtained from the Comparative Toxicogenomics Database (CTD). These gene sets were subsequently transformed into a one-hot matrix consisting of genes and gene set names for use as a knowledge matrix.

### Conversion of WGBS and RRBS data to HM450 probewise data

WGBS and RRBS data were converted to HM450 probewise data for comparative analysis with HM450 data using methyLiftover (https://github.com/Christensen-Lab-Dartmouth/methyLiftover)^26^ to compare with the HM450 probewise data. The original methyLiftover converts WGBS and RRBS data mapped to hg19 to HM450 probe-wise data; however, since the WGBS and RRBS data obtained from ENCODE were mapped to hg38, the program was slightly edited to convert them to HM450 probe-wise data using the hg38 remapped HM450 manifest file (http://zwdzwd.github.io/InfiniumAnnotation)^27^ created by Zhou et al.

### NMF with knowledge matrix sparse constraints

NMF with sparse regularization by a knowledge matrix was performed using the R PLIER package (https://github.com/wgmao/PLIER)^22^. With the PLIER package, we aimed to minimize the following equations by searching for matrices ***U***, ***Z***, and ***B*** when given an input data matrix ***D*** and a knowledge matrix ${\boldsymbol{C}}\in 0,{1}^{n\times m}$ consisting of m gene sets containing n genes, where ***U*** is the loading matrix for the knowledge matrix and ***B*** is the loading matrix for the latent feature matrix ***Z***:

$${\Vert {\boldsymbol{D}}-{\boldsymbol{ZB}}\Vert }_{F}^{2}+{\lambda }_{1}{\Vert {\boldsymbol{Z}}-{\boldsymbol{CU}}\Vert }_{F}^{2}+{\lambda }_{2}{\Vert {\boldsymbol{B}}\Vert }_{F}^{2}+{\lambda }_{3}{\Vert {\boldsymbol{U}}\Vert }_{{L}^{1}}$$

$$subject\,to\,{\boldsymbol{U}}\, >\, 0,\,{\boldsymbol{Z}}\, >\, 0$$

The first term is the degree of approximation index of the matrix product ***ZB*** of the latent feature matrix ***Z*** and the factor loading matrix ***B*** to the input data matrix ***D***, the so-called reconstruction error. The second, third and fourth terms are regularization terms for the matrices ***Z***, ***U***, and ***B***. Each regularization term is adjusted by the hyperparameters ${\lambda }_{1}$, ${\lambda }_{2}$, and ${\lambda }_{3}$. In addition, both matrices ***Z*** and ***U*** are non-negative matrices. In the second term, the matrix product ***CU*** of the knowledge matrix ***C*** and its factor loading matrix ***U***, given as constants, is intended to approximate the latent feature matrix ***Z***. A regularity condition due to the ${L}^{1}$ norm of the factor loading matrix ***U*** is given in the fourth term, which optimizes the latent feature matrix ***Z*** to be sparser. The third term is the Frobenius norm of the factor loading matrix ***B*** relative to the latent matrix ***Z***. The purpose is to ensure that the factor loading matrix ***B*** is not too large relative to the latent feature matrix ***Z***. In this study, PLIER was run on data ***D*** converted from probe-wise data to gene-wise data with the following hyperparameters: frac = 0.7, max.iter = 350, maxPath = 10, minGenes = 10, glm_alpha = 0.9, and tol = 10^−6^.

### Unsupervised clustering

Unsupervised clustering was performed to capture the macroscopic sample characteristics of the DNA methylation data and loading matrix output from methPLIER. Hierarchical cluster analysis (HCA), principal component analysis (PCA), k-means clustering, and uniform manifold approximation and projection (UMAP)^28^ were used for clustering. HCA performed clustering classification using Ward’s method^29^ based on the Euclidean distance between samples. k-means clustering was performed using the Hartigan‒Wong method^30^, with a maximum number of iterations of 100. UMAP was based on the Euclidean distance between samples and included the following parameters: neighbors = 15, components = 2, and epochs = 200.

### Motif analysis of sequences around DMPs

For the motif analysis of sequences surrounding differentially methylated positions (DMPs), we utilized the Analysis of Motif Enrichment (AME) tool included in the MEME Suite^31^. We focused on the 50 base pair genomic sequences corresponding to the probe design locations on the HumanMethylation BeadChip. To compare the hypomethylated and hypermethylated DMPs in pan-negative cases relative to those in *EGFR* mutation cases, we performed a one-tailed Fisher’s exact test for statistical evaluation. Motifs exhibiting significant differences (*P* < 0.05) were extracted for further analysis.

### Availability and implementation

The code used to construct methPLIER was deposited on GitHub (https://github.com/hamamoto-lab/methPLIER). Additionally, the data used for building methPLIER and the methPLIER model itself were deposited in Figshare (10.6084/m9.figshare.21938528, https://figshare.com/s/e656bd498a660778f988). By building a Docker image from the Dockerfile available on GitHub, users can create an analytical environment that enables the utilization of methPLIER.

### Software and packages

The analysis was performed using R 4.1.1 (https://cran.r-project.org), R Studio 1.3 (https://www.rstudio.com), and the following R packages: ChAMP^32^, minfi^33^, PLIER (https://github.com/wgmao/PLIER)^22^, multiPLIER (https://github.com/greenelab/multi-PLIER)^13^, and umap (https://github.com/tkonopka/umap).

## Results

### Development of methPLIER

**Fig. 2:**
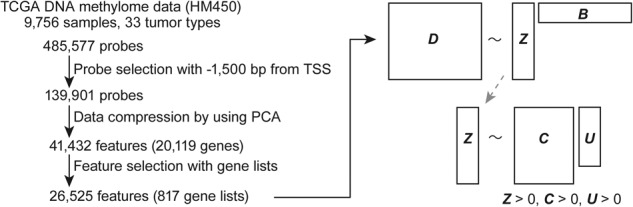
Diagram of methPLIER development. For the development of methPLIER, we used 9756 samples of Illumina Infinium HumanMethylation450 BeadChip (HM450) data obtained from the TCGA. We selected probes located within 1500 bps of the TSS. Next, we compressed the selected probe data to gene-wise data using principal component analysis (PCA) (for more detail, see Methods). The compressed gene-wise data were filtered by the gene list of the knowledge matrix, and we obtained the source domain data of methPLIER, comprising 26,525 features and 9756 samples. The source domain data were decomposed via non-negative matrix factorization with a knowledge matrix having sparse constraints. The details and equations used are provided in the Methods section.

To construct methPLIER, HM450 data from 9756 samples obtained from the TCGA were used (Fig. 2). methPLIER is an NMF performed under regular conditions where the latent matrix sparsely contains the knowledge matrix, which is a matrix consisting of multiple gene lists. The methodology of methPLIER presupposes a relationship between the input data and the prior knowledge matrix, which is necessary because of its reliance on a matrix decomposition approach based on this prior knowledge. This knowledge predominantly comprises curated information such as pathway information and ontology data, which are generally more compatible with gene expression data. However, a direct correlation between DNA methylation data and pathways or ontologies is not always evident, often leading to a lack of applicable prior knowledge. To address this challenge, innovative strategies for aligning analytical probes using knowledge matrices must be devised. This alignment is crucial for ensuring the relevance and accuracy of the matrix decomposition process in the context of DNA methylation data. In addition, the knowledge matrix used in our study was exclusively available at the gene level and not at the probe level. Since the knowledge matrix is a matrix consisting of multiple gene lists, HM450 data expressed as DNA methylation data for a single CpG site were converted into data for each gene to perform matrix decomposition. First, DNA methylation data for analysis probes located within 1500 bps of the TSS for each transcript were extracted and compressed for each transcript via PCA. Among the principal components obtained via PCA, the PCSs for the principal components reached the point where the cumulative contribution ratio exceeded 0.8 and were used as the compressed data for each transcript. The TCGA DNA methylation data were compressed into a matrix of 9756 samples ×26,525 features. NMF was subsequently performed on the compressed data under the regularization condition that the knowledge matrix was sparsely contained, resulting in a latent matrix ***Z*** with 524 LVs (Fig. 2). To use the methPLIER tool, matrix decomposition was performed on new DNA methylation data using this latent matrix Z to derive a loading matrix ***B***. The loading matrix for the feature space was represented by the same latent matrix ***Z***, allowing DNA methylation data from different datasets to be analyzed in a common feature space. Unsupervised clustering classification and intergroup comparative analysis can then be performed using this loading matrix.

### methPLIER enables analysis that reflects sample characteristics

**Fig. 3:**
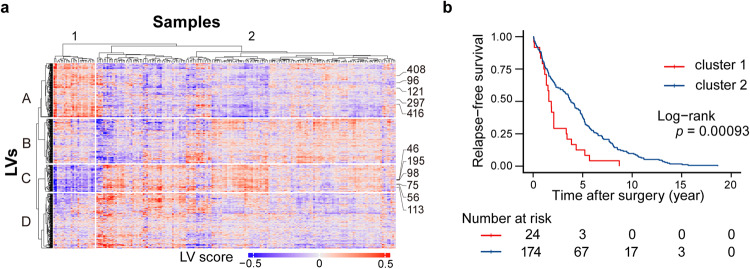
Results of lung adenocarcinoma dataset analysis using methPLIER. **a** Hierarchical cluster analysis (HCA) and heatmap plot of the loading matrix of the lung adenocarcinoma dataset (GSE39279). The columns and rows indicate samples and latent variables (LVs), respectively. The columns were divided into two clusters according to the HCA results. The rows were divided into four clusters via k-means clustering. **b** Kaplan‒Meier estimates for relapse-free survival (RFS) for the dataset with RFS information according to the two groups obtained from HCA clustering. The *p* value was calculated via the log-rank test.

To evaluate the performance of the methPLIER dataset, we analyzed a LUAD dataset (GSE39279)^15^ obtained from the GEO. The LUAD dataset was compressed into a matrix decomposable data format using eigenvectors obtained during the compression of TCGA DNA methylation data, followed by latent matrix decomposition. HCA was performed on the resulting loading matrix (Fig. 3a). Based on the HCA results, the samples were divided into two clusters, and Kaplan‒Meier survival analysis was performed for each cluster (Fig. 3b). The log-rank test results showed a significant difference in RFS between the two clusters (*p* = 0.00093). In a previous study using the same dataset, the sample clusters were divided into two groups based on the HCA results, and the results of the survival response analysis showed that there were two groups, one with a high recurrence risk and the other with a low recurrence risk. This grouping is consistent with the results of methPLIER analysis (GSE39279)^15^.

### Feasibility of using 1st exon region probes in methPLIER analysis

In the construction of methPLIER, a critical element is the use of a knowledge matrix primarily derived from gene-wise data, which extensively incorporates curated information on gene expression patterns in relation to pathways and ontologies. To compensate for the lack of scrutiny of the correlation of the knowledge matrix with DNA methylation patterns, the analysis concentrated on probes near the TSS, specifically within a 1500 base pair area known for its strong link to gene expression. To expand the scope of methPLIER further, we incorporated data compression for probes located in the 1st exon region, acknowledging the potential interest of researchers in this field. This decision was based on the assumption that DNA methylation in the 1st exon is likely to correlate with gene expression, which parallels the rationale applied to the TSS. The methodology was subsequently applied and validated using a lung cancer dataset. The results of the expanded analysis are discussed. By using factor loading matrix ***B*** for hierarchical clustering analysis in methPLIER constructed with 1st exon region data, we observed the formation of two distinct clusters, mirroring the findings from the TSS1500-based analysis (Supplementary Fig. 1). Crucially, survival time analysis based on cluster labels revealed significant differences between the two groups, underscoring the robustness of the approach.

### LVs obtained via methPLIER have high biological interpretability

Comparative analysis of LVs between the two clusters revealed significant differences in 261 LVs (FDR < 0.05, Supplementary Table 1). The knowledge variables in the top 10 LVs were confirmed by heatmap plotting, which showed that the knowledge matrices were sparse among the latent variables and included gene sets related to lung cancer and cancer progression, such as those related to KRAS, the p38 MAPK pathway, and the integrin pathway (Supplementary Fig. 2). A heatmap of the contents of the knowledge variables in the top 10 LVs showed that the knowledge matrices were sparsely included among the latent variables. LV96 contained a set of genes whose expression was suppressed when the oncogenic form of KRAS (G12V) was overexpressed in four epithelial cell types^34^, and LV121 contained the p38 MAPK signaling pathway, all of which scored higher in the short-term RFS group. Lung cancer patients with *KRAS* G12V mutations have been shown to have shorter overall survival than non-*KRAS* G12V mutation cases^35^. In addition, p38 generally functions as a tumor suppressor protein; however, in vitro experiments have shown that it contributes to cell proliferation and malignant transformation in transgenic lung cancer cell lines harboring the *KRAS* G12V mutation^36^. Based on the above, we believe that latent features in methPLIER reflect biological characteristics and allow DNA methylation data analysis with high explanatory potential.

### methPLIER reduces preprocessing method-associated differences in data distribution

**Fig. 4:**
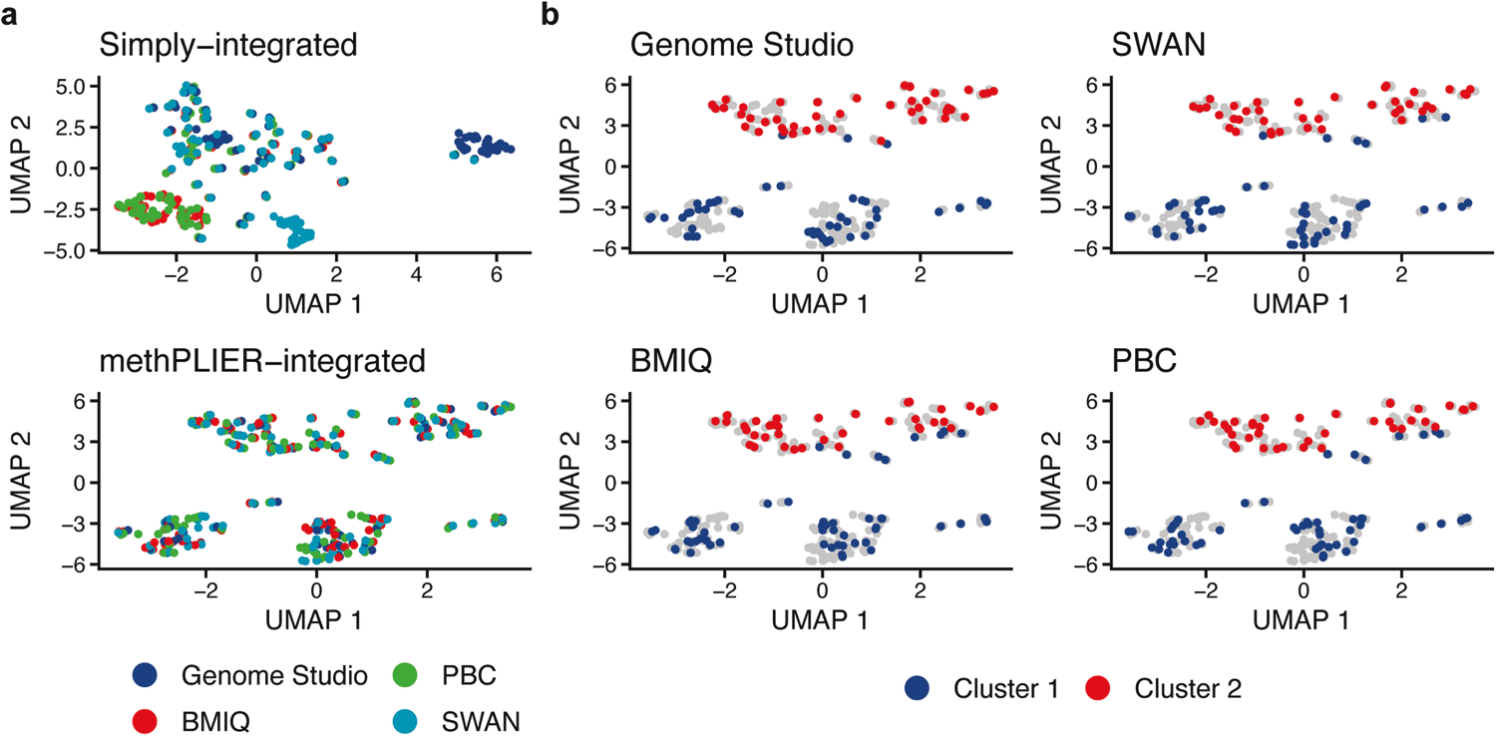
Comparison of the UMAP plot of the GSE61441 dataset preprocessed using various preprocessing methods. **a** UMAP plot of integrated data preprocessed using various methods. The data were preprocessed at each time point through the following methods: Genome Studio (blue), SWAN (light blue), BMIQ (red), and PBC (green). The upper panel shows the UMAP plot of the data, which were integrated without using methPLIER, and the lower panel shows the UMAP plot of the integrated data obtained using methPLIER. **b** UMAP plot of data preprocessed via each method: mimicked method of Genome Studio (upper left), SWAN (upper right), BMIQ (lower left), and PBC (lower right). The red and blue points indicate the respective clusters, and the gray points indicate the data obtained using other preprocessing methods.

To examine the effect of methPLIER on data distribution bias due to differences in preprocessing methods, dataset distribution comparisons were performed using a single dataset preprocessed via four different preprocessing methods. The dataset comprised 92 renal cell carcinoma samples and was preprocessed via four methods: a preprocessing method mimicking the Illumina Genome Studio preprocessing method (Genome Studio)^33^, subset-quantile within array normalization (SWAN)^37^, peak-based correction (PBC)^38^, and beta-MIxture Quantile dilation (BMIQ)^39^. The data preprocessed via each method were then combined into simply integrated data and into data with the loading matrix derived by methPLIER (methPLIER-integrated). Each of these merged datasets was embedded into a two-dimensional space via UMAP, and a color-coded scatter plot was generated for each preprocessing method. After simple integration, clusters were formed for each preprocessing method, while after using methPLIER for analyses, no clusters were observed (Fig. 4). The methPLIER-integrated data were then classified into two clusters by k-means clustering and color coded based on the classification results. In both preprocessing methods, the respective clusters were separated on the UMAP 2 axis, showing a similar pattern regardless of the preprocessing method (Fig. 4b). Color coding of the UMAP plots by sample type (cancer/noncancer) and sex information for the dataset used in the analysis showed a similar pattern to that of the cluster information obtained by K-means clustering (Supplementary Fig. 3). These results suggest that methPLIER reduces the data distribution bias caused by the preprocessing method.

### methPLIER reduces differences in data distribution across analysis platforms

**Fig. 5:**
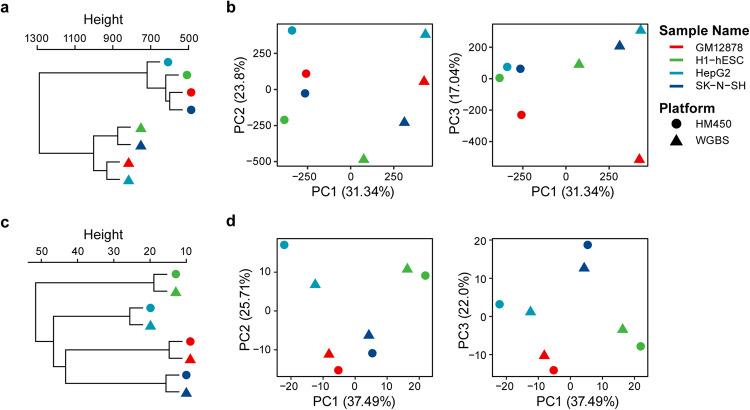
Results of unsupervised classification of combined data with and without methPLIER. Validation of the performance of the methPLIER tool on DNA methylation data acquired via different platforms HCA and PCA plot of the data integrated with (lower: **c**, **d**) or without (upper: **a**, **b**) using methPLIER. The colors of the points indicate the sample names: GM12878 (red), H1-hESC (green), HepG2 (light blue), and SK-N-SH (blue). The shape of the points indicates the type of platform used to acquire the data: HM450 (circle) or WGBS (triangle).

DNA methylation data can be obtained not only via BeadChip analysis but also via NGS-based WGBS. WGBS has been increasingly used in recent years due to its ability to yield genome-wide methylation data, including data on DNA methylation at non-CpG sites. Therefore, to investigate the potential of using methPLIER for WGBS data analysis, we analyzed data from four cell lines in the ENCODE project via the HM450K and WGBS databases. Since methPLIER is an analysis method compatible with HM450K probe data, WGBS data were converted to corresponding HM450K analysis probe data using MhyLiftover^26^. The converted WGBS data were merged with the HM450K data obtained from the same cell line, and unsupervised clustering was performed via HCA to form clusters for each analysis platform (Fig. 5a). PCA was performed on the integrated data, and a scatter plot of principal components (PCs) 1 to 3 was generated, revealing that clusters were formed for each analysis platform, as was the case for HCA (Fig. 5b). This finding suggested that direct integration of WGBS data with HM450K data is subject to data distribution bias due to the analysis platform. The loading matrix was subsequently obtained using methPLIER for the previously integrated data, with classification by HCA and PCA showing that clusters were formed based on cell lines (Fig. 5c, d) rather than analysis platforms, as previously observed. These results suggest that the loading matrix output by methPLIER reduces the data distribution bias caused by differences in analysis platforms and enables an analysis that focuses on sample characteristics. In addition, we evaluated the suitability of the methPLIER tool for use with RRBS data. The results showed that the RRBS data formed distinct clusters (Supplementary Fig. 4a–d). This pattern was attributable to the limited alignment of the RRBS data with the HM450K probes, which achieved only an approximately 10% match, in contrast to the higher compatibility observed for WGBS data (Supplementary Fig. 4e). Despite the use of methPLIER, this discrepancy led to the persistent formation of separate clusters in the RRBS data, highlighting a notable platform-specific bias.

### The knowledge matrix of methPLIER can be flexibly modified for different purposes

Since the latent feature matrix of methPLIER is constrained by the knowledge matrix, the biological interpretation of latent features is strongly influenced by the knowledge matrix applied in constructing methPLIER. The methPLIER tool constructed thus far used classical pathways and cancer-related gene sets focused on cell biological functions for general-purpose data analysis. However, in drug response prediction and drug development research, we expect that knowledge matrices, which include gene sets, such as pathways and ontologies related to compounds, and drugs that can be easily analyzed at a later stage and easily explained to stakeholders, will be more appropriate. Therefore, we reconstructed methPLIER using the adenocarcinoma-related gene set obtained from the CTD as the knowledge matrix and examined the variability of the knowledge matrix in methPLIER and its usefulness. The CTD is a database that aggregates the effects of environmental substances, compounds, and drugs on living organisms, with curated toxicogenomic data available for more than 16,300 compounds. In this study, among adenocarcinoma-related chemicals in the CTD, we extracted the inference network that had more than 10 reference papers, created a knowledge matrix consisting of 2545 chemicals and 778 genes, and constructed a methPLIER-CTD with 230 LVs.

### PRISM DNA methylation dataset analysis using the methPLIER-CTD

**Fig. 6:**
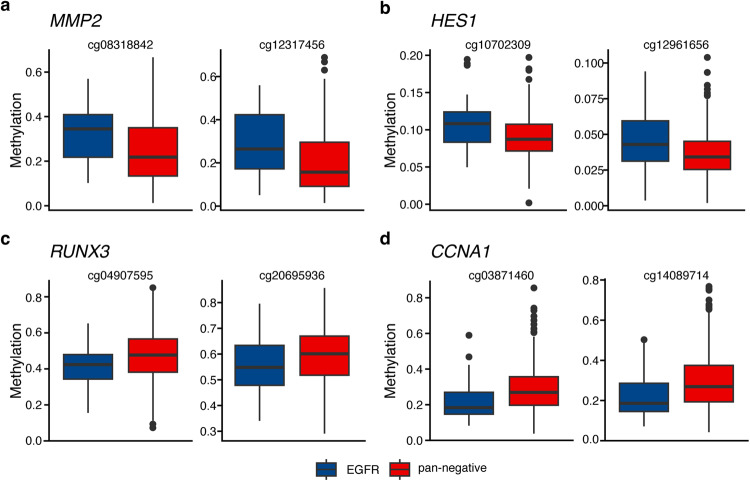
Box-and-whisker diagram of DMPs in the anticancer drug-related gene set obtained using methPLIER-CTD. The vertical axis shows the DNA methylation level in each DMP, and the color of each box whisker indicates *EGFR* mutation cases in blue and pan-negative cases in red. **a**, **b** DMPs showing significant hypomethylation in pan-negative cases. **c**, **d** DMPs showing significantly greater methylation in pan-negative cases.

**Fig. 7:**
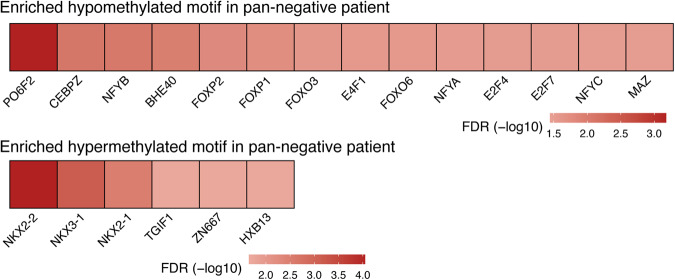
Heatmap of motif analysis for sequences surrounding DMPs. The heatmap displays the transcription factor binding motifs that were enriched in hypomethylated DMPs (upper figure) and hypermethylated DMPs (lower figure) in pan-negative cases compared to *EGFR* mutation cases. The color of the heatmap represents the -log10 value of the false discovery rate (FDR).

The methPLIER-CTD was used to analyze the DNA methylation dataset of lung cancer patients in the PRISM database. It has been reported that approximately half of Japanese lung adenocarcinoma patients have *EGFR* mutations, and approximately 30% of patients have no identified driver genes, such as *KRAS*, *ALK* fusion, or *RET* fusion genes (pan-negative)^40^. A two-group comparative analysis of *EGFR*-mutated and pan-negative cases was performed on the PRISM DNA methylation dataset generated by using the methPLIER-CTD. A *t* test for factor loadings on each latent feature obtained via methPLIER-CTD was performed, and LVs with an FDR of less than 0.05 according to the Benjamini–Hochberg method were extracted. The results revealed significant differences in 92 LVs, and 64 features contained gene sets related to lung cancer (Supplementary Table 2). To confirm the biological interpretability of these 64 LVs, a heatmap plot of the factor loadings on the gene set for each latent feature was generated (Supplementary Fig. 5). Each latent feature contained sparse gene sets, including cancer-related gene sets, anticancer drug-related gene sets, general drug-related gene sets, toxicant-related gene sets, endogenous molecule-related gene sets, and other suggestive gene sets. Focusing on the anticancer drug-related gene set, 54 LVs were extracted. Comparison of the DNA methylation levels of analytical probes located near genes in the anticancer drug-related gene set in *EGFR*-mutated patients and pan-negative cases revealed 841 DMPs located near 225 genes (FDR < 0.05, Supplementary Table 3). Among the DMPs, 466 DMPs tended to be hypomethylated in pan-negative cases, while 375 DMPs tended to be hypermethylated. The genes that exhibited hypomethylation in pan-negative cases included *MMP2*^41,42^, which has been reported to be involved in metastasis and local invasion in breast cancer, and *HES1*^43^, which has been reported to be hypomethylated in colorectal cancer (Fig. 6a, b). Among the genes that showed a trend toward hypermethylation in the pan-negative cases were *RUNX3*, a known tumor suppressor gene, and *CCNA1*^44^, which has been reported to correlate with treatment response to doxorubicin and 5-fluorouracil (Fig. 6c, d). We performed transcription factor binding motif analysis on the 50 surrounding bps of the 466 identified hypomethylated DMPs and 375 hypermethylated DMPs (Fig. 7). Among the hypomethylated DMPs, we observed enrichment of transcription factor binding motifs for POU Class 6 Homeobox 2 (PO6F2), CCAAT Enhancer Binding Protein Zeta (CEBPZ), Nuclear Transcription Factor Y Subunit Beta (NFYB), Basic Helix-Loop-Helix Family Member E40 (BHE40), Forkhead Box P2 (FOXP2), Forkhead Box P1 (FOXP1), Forkhead Box O3 (FOXO3), E4F Transcription Factor 1 (E4F1), Forkhead Box O6 (FOXO6), NFYA, E4F Transcription Factor 1 (E2F4), E2F Transcription Factor 7 (E2F7), Nuclear Transcription Factor Y Subunit Gamma (NFYC), and MYC Associated Zinc Finger Protein (MAZ). In contrast, hypermethylated DMPs displayed enrichment of transcription factor binding motifs for NK2 homeobox 2 (NKX2-2), NK3 homeobox 1 (NKX3-1), NK2 homeobox 1 (NKX2-1), TGFB Induced Factor Homeobox 1 (TGIF), Zinc Finger Protein 667 (ZN667), and Homeobox B13 (HXB13). NKX2-1, an airway epithelial-specific transcription factor, has been shown to inhibit SPDEF expression, effectively preventing ovalbumin-induced goblet cell differentiation and lung inflammation in transgenic overexpression of Nkx2-1^45^. Intriguingly, in the context of regulating its target genes surfactant protein B and myosin-binding protein H, NKX2-1 has been found to be sensitive to methylation^46–48^. This finding contributes to a deeper understanding of the molecular mechanisms involved in airway epithelial cell regulation and may have implications for future research in this area. In the pan-negative cases, we observed enrichment of NKX2-1 recognition sequences in the highly methylated DNA regions. Based on these findings, it is plausible that in pan-negative cases, the binding of NKX2-1 and NKX family proteins may be hindered by high DNA methylation. This could result in insufficient suppression of cancer-related gene expression, potentially leading to the onset and progression of cancer. In summary, data analysis using methPLIER-CTD constructed with drug-related gene sets revealed highly methylated regions near drug response-related genes and tumor suppressor genes in pan-negative cases.

## Discussion

DNA methylation patterns are correlated with cell and tissue type as well as with disease and have been applied for diagnosis and therapy response prediction under various conditions^49^. In studies of rare and regional diseases, it is important to integrate DNA methylation data from multiple studies, as small sample sizes and limitations inherent to data from a single institution or study may compromise analysis^50,51^. However, DNA methylation data in various public databases differ according to the platform employed and processing applied, which constitutes a barrier to data integration. Therefore, we developed a new DNA methylation data analysis method, namely, methPLIER, which is oriented toward integrated data analysis. Although methPLIER is inspired by MultiPLIER, it is not a mere adaptation for DNA methylation data analysis. methPLIER has three novel aspects compared to MultiPLIER and other existing methods. First, methPLIER employs a unique preprocessing method for the input data. The DNA methylation data used by methPLIER represents the methylation rate of each CpG site, resulting in a “one-to-many relationship”, where multiple methylation information exists for a single gene or transcript. In contrast, the knowledge matrix utilized for regularization comprises gene-specific matrices. Consequently, transforming CpG site data into gene-specific data is essential. methPLIER compresses the DNA methylation data for each transcript by extracting the methylation data for analysis probes situated within 1500 bps of the transcript’s transcription start site and compressing them using principal component analysis^52,53^. Although DNA methylation is considered an epigenetic modification that represses expression^54,55^, in cancer cells and stem cells, a hypermethylated state around the promoter contributes to or correlates with expression activity^56–58^. For example, in the *TERT* gene, the methylated variable region is located 1000 bps upstream from the TSS, and the 500 bps upstream region near the TSS was reportedly hypomethylated, regardless of expression^56,57^. Gu et al. examined the association between DNA methylation and the expression of promoter regions in 265 samples of clear cell renal cell carcinoma (ccRCC) and 133 adjacent tissues from The Cancer Genome Atlas (TCGA)^59^. They analyzed TSS200, TSS1500, and 5’UTR sites as promoters and found that of the regions with ccRCC-specific methylation patterns, 81.81% were TSS200 sites, 6.97% were TSS1500 sites, and 11.22% were 5’UTRs. Nearly 90% of the specific methylation variation CpGs were located within 1500 bases around the TSS. Analysis of the association between specific methylation variations in promoters and gene expression showed that 31.2% of the promoters were associated with expression variation. Therefore, compressing DNA methylation data down to the 1500-bp upstream region, as a simple mean or median, may lead to distal methylation patterns that are relevant to expression being overlooked. We employed PCSs for principal components whose cumulative contribution ratio exceeded 0.8 as the compressed data for each transcript. This approach was chosen for three reasons: many variable DNA methylation regions related to gene expression regulation are located near transcription start sites; gene expression regulation through DNA methylation is often governed by differences in the genomic patterns of neighboring regions rather than variations in methylation patterns at individual CpG sites; and DNA methylation patterns near the transcription start site have been reported to modulate transcriptional variant expression patterns. Second, methPLIER can integrate and analyze data acquired from different platforms or research teams while reducing data distribution bias. BeadChip analysis was developed more than a decade ago and has been analyzed by many research teams, making its vast datasets valuable research tools. However, there are significant differences in the data distribution bias between WGBS data and other datasets, and the data characteristics can be masked by this large bias. In our study, we used the latent feature matrix for approximately 10,000 samples to perform matrix factorization of the analysis target sample, reducing the bias between datasets and platforms. This enabled us to utilize accumulated valuable resources in future analyses without wasting them and could be useful for meta-analyses of rare diseases where it is difficult to collect sufficient cases at a single facility. Finally, methPLIER is unique in that interpreting the results after data analysis is straightforward. In traditional DNA methylation data analysis, variable sites or variable regions are obtained by comparative analysis, followed by functional estimation analysis, such as pathway analysis, GO analysis, or gene set enrichment analysis. On the other hand, methPLIER performs matrix factorization using the latent matrix ***Z***, which sparsely includes the knowledge matrix, and the loading matrix ***B*** outputted by methPLIER contains information about the knowledge matrix. Therefore, it is possible to perform functional estimation analyses, such as pathway analysis, GO analysis, and data feature analysis, simultaneously. This broadens the gateway to DNA methylation data analysis and is useful for users unfamiliar with data analysis. In unsupervised clustering analysis using factor loadings on latent characteristic variables derived via methPLIER, the results reflect sample characteristics, such as progression-free survival and cell characteristics, highlighting its utility for DNA methylation data analysis. Furthermore, the factor loading matrix derived by methPLIER reduces the data distribution bias for datasets generated with different platforms and preprocessing methods, which is important for dataset-to-dataset integration analysis.

The methPLIER-CTD, which was constructed by modifying the methPLIER knowledge matrix and oriented toward drug-related DNA methylation analysis, may have demonstrated the usefulness of methPLIER for purpose-oriented analysis. Comparative analysis of methPLIER-CTD between pan-negative and *EGFR*-mutated lung cancer patients identified 841 lung cancer pan-negative case-specific DMPs associated with the drug-related gene set and genomic regions of interest. *CCNA1*, which is hypermethylated according to methPLIER, contributes to cell cycle regulation and was previously reported to be hypermethylated around its promoter in cervical and breast cancer tumors^44,60^. In addition, DNA methylation patterns around promoters have been reported to be associated with the sensitivity of breast cancer cells to doxorubicin and 5-fluorouracil^44^. *RUNX3*, which is hypermethylated in pan-negative cases, is known to function as a tumor suppressor gene that inhibits Wnt signaling by inhibiting β-catenin/Tcfs, and its expression is known to be regulated by DNA methylation^61^. Since *RUNX3* is reportedly hypermethylated in various cancer types, including lung cancer^62,63^, we speculate that Wnt inhibitors, such as vantictumab and LGK974, which are currently in clinical trials for other cancer types, may be useful in the treatment of pan-negative cases.

Although we believe that the methPLIER tool is useful for analyzing DNA methylation data, there are several limitations. First, the biological interpretability of the latent feature matrix ***Z*** in methPLIER varies greatly depending on the knowledge matrix used to construct methPLIER. Therefore, when discussing the results of the analysis using the factor loading matrix ***B*** derived by methPLIER, it is necessary to consider what knowledge matrix the methPLIER used in the analysis. On the other hand, this is both a caveat in using methPLIER and an advantage in objective-oriented data analysis, such as the example of PRISM DNA methylation dataset analysis using methPLIER-CTD, which was reconstructed using CTD. Second, methPLIER uses data from only a portion of the region in the BeadChip. methPLIER compresses probe-wise data to gene-wise data prior to matrix decomposition. These data were compressed by principal component analysis (PCA) using analysis probes located 1500 bases upstream from the TSS, thus eliminating DNA methylation data located in introns and intergenic regions. Therefore, when expectedly analyzing DNA methylation status at a distance away from the TSS, it is necessary to reconstruct the methPLIER gene under different probe conditions for compression. The third limitation is that the results of the analysis obtained by methPLIER do not guarantee the regulation of gene expression by DNA methylation. As mentioned above, methPLIER analyzes methylation data around promoters, and it is highly likely that the results contribute to the regulation of gene expression. On the other hand, the regulation of gene expression via DNA methylation is still unclear in many respects, and whether the DNA methylation patterns observed between samples are directly involved in the regulation of expression must be confirmed separately by gene expression data or wet laboratory experiments. Fourth, the results of the methPLIER analysis were influenced by variations in tumor content among the samples. This issue arose because methPLIER is designed to analyze preprocessed data without an inherent mechanism for adjusting for tumor content. Consequently, disparities in tumor content could influence the analysis results. Nonetheless, it is important to consider that the TCGA data used for developing methPLIER not only encompassed tumor regions but also included benign tissues and represented cancers from various tissues. This diverse composition suggests that the latent features identified by methPLIER are likely to capture characteristics specific to different tissues and cell types. When the tumor content varied between samples, we predicted that methPLIER would tend to return results enriched with latent features not directly related to the tumor. This can indirectly indicate potential variations in tumor content across samples. These insights can direct users toward the application of specialized methods that have been developed to estimate tumor content, allowing for more targeted correction of their data. Despite these limitations, we believe that methPLIER will increase the interpretability of DNA methylation analysis and enable easy and comprehensive analyses, including dataset comparative analysis and ontology analysis. The use of methPLIER will also enable cross-dataset analysis of DNA methylation data in medicine while maintaining biological interpretability, facilitate meta-analysis in rare disease and epidemiological studies, and accelerate the discovery of disease-related DNA methylation patterns and treatment effect-related DNA methylation patterns.

The advent of numerous omics data analysis pipeline tools has significantly advanced the field of bioinformatics by streamlining complex data analysis processes^32,33,64–66^. These tools integrate a variety of functions, from raw data ingestion to normalization, batch normalization, and comparative analysis, within a single-command framework. The incorporation of graphical user interfaces in some of these tools has democratized access to data analysis, enabling researchers with limited programming expertise to engage in sophisticated analytical processes^65,66^. Despite their utility, these pipeline tools often require substantial computational resources, including multicore CPUs and extensive memory, particularly when processing raw data files, such as FASTQ or IDAT files. This demand poses a barrier, necessitating significant computational infrastructure and extended processing times. In contrast, methPLIER operates differently, focusing on the analysis of preprocessed matrix files rather than raw data. Positioned downstream in the analytical pipeline, methPLIER can use the lighter preprocessed data typically available in public repositories, such as GEO, or as supplementary data in academic publications. This methodological shift offers a pragmatic approach for preliminary data analysis, enabling rapid and cost-effective insights that are particularly valuable in the early stages of research. Comprehensive integrated comparative analyses generally require high-end computational resources and standardized data analysis procedures facilitated by established pipeline tools. However, this approach is not without drawbacks, including considerable financial, temporal, and labor costs, without guaranteed analytical success. Therefore, methPLIER is a strategic alternative. Enabling preliminary integrated analyses allows researchers to acquire initial data quickly and affordably, thus facilitating a more cost-effective approach to integrated data analysis. In summary, the purpose of using methPLIER in the broader analytical framework is not to replace more resource-intensive pipeline tools but rather to complement them. By offering a means to conduct efficient preliminary analyses, methPLIER enhances the overall workflow and balances the depth and breadth of the analysis against the practical constraints of resource allocation. This approach is particularly relevant in the context of high-throughput omics studies, where the initial screening of vast datasets is as crucial as a detailed analysis.

In conclusion, our new DNA methylation data analysis tool, methPLIER, has high biological interpretability, and the knowledge matrix can be modified according to the purpose of the analysis. Moreover, this approach reduces the data bias between datasets caused by differences in preprocessing methods and analysis platforms, contributes to highly reproducible data analysis and facilitates integrated analysis between datasets. In the future, the expansion of DNA methylation data analysis using methPLIER will contribute to the elucidation of the true nature of pathologies associated with DNA methylation abnormalities and the promotion of the search for disease-related markers supported by biological significance.

### Supplementary information

Supplementary information

Supplementary Table 1

Supplementary Table 2

Supplementary Table 3

## Data Availability

The data are available upon reasonable request.

## References

[1] Zemach A, McDaniel IE, Silva P, Zilberman D (2010). Genome-wide evolutionary analysis of eukaryotic DNA methylation. Science.

[2] Greenberg MVC, Bourc’his D (2019). The diverse roles of DNA methylation in mammalian development and disease. Nat. Rev. Mol. Cell Biol..

[3] Kulis, M & Esteller, M. DNA Methylation and Cancer. *Adv. Genet.* **70**, 27–56 (2010). 10.1016/B978-0-12-380866-0.60002-2 20920744

[4] Plongthongkum N, Diep DH, Zhang K (2014). Advances in the profiling of DNA modifications: cytosine methylation and beyond. Nat. Rev. Genet.

[5] Hamamoto R, Komatsu M, Takasawa K, Asada K, Kaneko S (2019). Epigenetics Analysis and Integrated Analysis of Multiomics Data, Including Epigenetic Data, Using Artificial Intelligence in the Era of Precision Medicine. Biomolecules.

[6] Naumov VA (2013). Genome-scale analysis of DNA methylation in colorectal cancer using Infinium HumanMethylation450 BeadChips. Epigenetics.

[7] Fasanelli F (2015). Hypomethylation of smoking-related genes is associated with future lung cancer in four prospective cohorts. Nat. Commun..

[8] Wang Z (2020). Epigenomic analysis of 5-hydroxymethylcytosine (5hmC) reveals novel DNA methylation markers for lung cancers. Neoplasia.

[9] Pidsley R (2013). A data-driven approach to preprocessing Illumina 450 K methylation array data. BMC Genomics.

[10] Felling, RJ, Guo, JU & Song, H Neuronal activation and insight into the plasticity of DNA methylation. *Epigenomics* **4**, 125–127 (2012). 10.2217/epi.12.2 22449183

[11] McEwen LM (2018). Systematic evaluation of DNA methylation age estimation with common preprocessing methods and the Infinium MethylationEPIC BeadChip array. Clin. Epigenetics.

[12] Collado-Torres, L et al. Reproducible RNA-seq analysis using recount2. *Nat. Biotechnol.* **35**, 319–321 (2017). 10.1038/nbt.3838 PMC6742427 28398307

[13] Taroni JN (2019). MultiPLIER: A Transfer Learning Framework for Transcriptomics Reveals Systemic Features of Rare Disease. Cell Syst..

[14] Hamamoto, R et al. Application of non-negative matrix factorization in oncology: one approach for establishing precision medicine. *Brief Bioinform* **23**, bbac246 (2022). 10.1093/bib/bbac246 PMC9294421 35788277

[15] Sandoval J (2013). A prognostic DNA methylation signature for stage I non-small-cell lung cancer. J. Clin. Oncol..

[16] Barrett T (2013). NCBI GEO: archive for functional genomics data sets–update. Nucleic Acids Res..

[17] Wei J-H (2015). A CpG-methylation-based assay to predict survival in clear cell renal cell carcinoma. Nat. Commun..

[18] Davis CA (2018). The Encyclopedia of DNA elements (ENCODE): data portal update. Nucleic Acids Res..

[19] Subramanian A (2005). Gene set enrichment analysis: a knowledge-based approach for interpreting genome-wide expression profiles. Proc. Natl Acad. Sci. USA.

[20] Liberzon A (2011). Molecular signatures database (MSigDB) 3.0. Bioinformatics.

[21] Liberzon A (2015). The Molecular Signatures Database (MSigDB) hallmark gene set collection. Cell Syst..

[22] Mao W, Zaslavsky E, Hartmann BM, Sealfon SC, Chikina M (2019). Pathway-level information extractor (PLIER) for gene expression data. Nat. Methods.

[23] Kanehisa M, Sato Y, Kawashima M, Furumichi M, Tanabe M (2016). KEGG as a reference resource for gene and protein annotation. Nucleic Acids Res..

[24] Abbas AR, Wolslegel K, Seshasayee D, Modrusan Z, Clark HF (2009). Deconvolution of blood microarray data identifies cellular activation patterns in systemic lupus erythematosus. PLoS One.

[25] Sturm D (2016). New Brain Tumor Entities Emerge from Molecular Classification of CNS-PNETs. Cell.

[26] Titus AJ, Houseman EA, Johnson KC, Christensen B (2016). C. methyLiftover: cross-platform DNA methylation data integration. Bioinformatics.

[27] Zhou W, Laird PW, Shen H (2017). Comprehensive characterization, annotation and innovative use of Infinium DNA methylation BeadChip probes. Nucleic Acids Res..

[28] McInnes L, Healy J, Saul N, Großberger L (2018). UMAP: Uniform Manifold Approximation and Projection. J. Open Source Softw..

[29] Ward JH (1963). Hierarchical Grouping to Optimize an Objective Function. J. Am. Stat. Assoc..

[30] Hartigan JA, Wong MA (1979). A k-means clustering algorithm. Appl Stat..

[31] McLeay RC, Bailey TL (2010). Motif Enrichment Analysis: a unified framework and an evaluation on ChIP data. BMC Bioinforma..

[32] Tian Y (2017). ChAMP: updated methylation analysis pipeline for Illumina BeadChips. Bioinformatics.

[33] Aryee MJ (2014). Minfi: a flexible and comprehensive Bioconductor package for the analysis of Infinium DNA methylation microarrays. Bioinformatics.

[34] Barbie DA (2009). Systematic RNA interference reveals that oncogenic KRAS-driven cancers require TBK1. Nature.

[35] Renaud S (2015). Prognostic value of the KRAS G12V mutation in 841 surgically resected Caucasian lung adenocarcinoma cases. Br. J. Cancer.

[36] Vitos-Faleato J (2020). Requirement for epithelial p38α in KRAS-driven lung tumor progression. Proc. Natl Acad. Sci. USA.

[37] Maksimovic J, Gordon L, Oshlack A (2012). SWAN: Subset-quantile within array normalization for illumina infinium HumanMethylation450 BeadChips. Genome Biol..

[38] Dedeurwaerder S (2011). Evaluation of the Infinium Methylation 450 K technology. Epigenomics.

[39] Teschendorff AE (2013). A beta-mixture quantile normalization method for correcting probe design bias in Illumina Infinium 450 k DNA methylation data. Bioinformatics.

[40] Shiraishi K (2016). Association of variations in HLA class II and other loci with susceptibility to EGFR-mutated lung adenocarcinoma. Nat. Commun..

[41] Artacho-Cordón F (2012). Matrix metalloproteinases: potential therapy to prevent the development of second malignancies after breast radiotherapy. Surg. Oncol..

[42] Pereira IT (2014). Fibronectin affects transient MMP2 gene expression through DNA demethylation changes in non-invasive breast cancer cell lines. PLoS One.

[43] Wu Y (2017). The clinicopathological significance of HES1 promoter hypomethylation in patients with colorectal cancer. Onco Targets Ther..

[44] Klajic J (2014). DNA methylation status of key cell-cycle regulators such as CDKNA2/p16 and CCNA1 correlates with treatment response to doxorubicin and 5-fluorouracil in locally advanced breast tumors. Clin. Cancer Res..

[45] Maeda Y (2011). Airway epithelial transcription factor NK2 homeobox 1 inhibits mucous cell metaplasia and Th2 inflammation. Am. J. Respir. Crit. Care Med..

[46] Cao Y (2010). Epigenetic mechanisms modulate thyroid transcription factor 1-mediated transcription of the surfactant protein B gene. J. Biol. Chem..

[47] Hosono Y (2012). MYBPH, a transcriptional target of TTF-1, inhibits ROCK1, and reduces cell motility and metastasis. EMBO J..

[48] Song J (2017). Aberrant DNA methylation and expression of SPDEF and FOXA2 in airway epithelium of patients with COPD. Clin. Epigenetics.

[49] Locke WJ (2019). DNA Methylation Cancer Biomarkers: Translation to the Clinic. Front. Genet..

[50] Sadikovic, B et al. Correction: Clinical epigenomics: genome-wide DNA methylation analysis for the diagnosis of Mendelian disorders. *Genet. Med.* **23**, 2228 (2021). 10.1038/s41436-021-01130-z PMC9119201 33637969

[51] Paparella A (2022). Genome-wide DNA methylation profiling and exome sequencing resolved a long-time misdiagnosed case. J. Hum. Genet.

[52] Moore LD, Le T, Fan G (2013). DNA methylation and its basic function. Neuropsychopharmacology.

[53] He X-J, Chen T, Zhu J-K (2011). Regulation and function of DNA methylation in plants and animals. Cell Res..

[54] Witte T, Plass C, Gerhauser C (2014). Pan-cancer patterns of DNA methylation. Genome Med..

[55] Esteller M (2002). CpG island hypermethylation and tumor suppressor genes: a booming present, a brighter future. Oncogene.

[56] Takasawa K (2018). DNA hypermethylation enhanced telomerase reverse transcriptase expression in human-induced pluripotent stem cells. Hum. Cell.

[57] Lee DD (2019). DNA hypermethylation within TERT promoter upregulates TERT expression in cancer. J. Clin. Invest..

[58] Rauluseviciute I, Drabløs F, Rye MB (2020). DNA hypermethylation associated with upregulated gene expression in prostate cancer demonstrates the diversity of epigenetic regulation. BMC Med Genom..

[59] Gu Y (2017). Promoter DNA methylation analysis reveals a novel diagnostic CpG-based biomarker and RAB25 hypermethylation in clear cell renel cell carcinoma. Int J. Biol. Sci. Rep..

[60] Zuo Q (2014). Methylation in the promoters of HS3ST2 and CCNA1 genes is associated with cervical cancer in Uygur women in Xinjiang. Int J. Biol. Markers.

[61] Wang Y (2014). Association of promoter methylation of RUNX3 gene with the development of esophageal cancer: a meta analysis. PLoS One.

[62] Kim TY (2004). Methylation of RUNX3 in various types of human cancers and premalignant stages of gastric carcinoma. Lab. Invest..

[63] Sato K (2006). Epigenetic inactivation of the RUNX3 gene in lung cancer. Oncol. Rep..

[64] Morris TJ, Beck S (2015). Analysis pipelines and packages for Infinium HumanMethylation450 BeadChip (450k) data. Methods.

[65] Assenov Y (2014). Comprehensive analysis of DNA methylation data with RnBeads. Nat. Methods.

[66] Müller F (2019). RnBeads 2.0: comprehensive analysis of DNA methylation data. Genome biol..

