# Supplementary material for: Advances in cancer DNA methylation analysis with methPLIER: use of non-negative matrix factorization and knowledge-based constraints to enhance biological interpretability
Source: Exp Mol Med. 2024 Mar 4;56(3):646–55. doi: 10.1038/s12276-024-01173-7 (PMC10985003; doi:10.1038/s12276-024-01173-7)
Supplement: Supplementary file 1 — Supplementary information [file 12276_2024_1173_MOESM1_ESM.pdf]

Supplementary information

Advances in cancer DNA methylation analysis with methPLIER: Use of non-negative matrix factorization and knowledge-based constraints to enhance biological interpretability

The file contains

Supplementary Figures 1 – 5

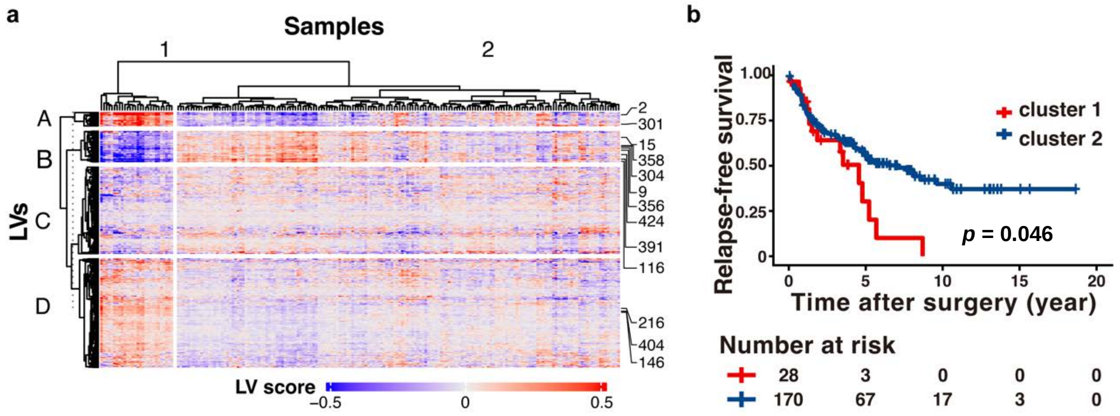

Supplementary Fig. 1. Results of lung adenocarcinoma dataset analysis using methPLIER constructed with 1st exon probes. (**a**) Hierarchical cluster analysis (HCA) and heatmap plot of the loading matrix of the lung adenocarcinoma dataset (GSE39279). The columns and rows indicate samples and latent variables (LVs), respectively. The columns were divided into two clusters according to the HCA results. The rows were divided into four clusters via k-means clustering. (**b**) Kaplan-Meier estimates for relapse-free survival (RFS) for the dataset with RFS information, according to the two groups obtained from HCA clustering. The *p*-value was calculated using a log-rank test.

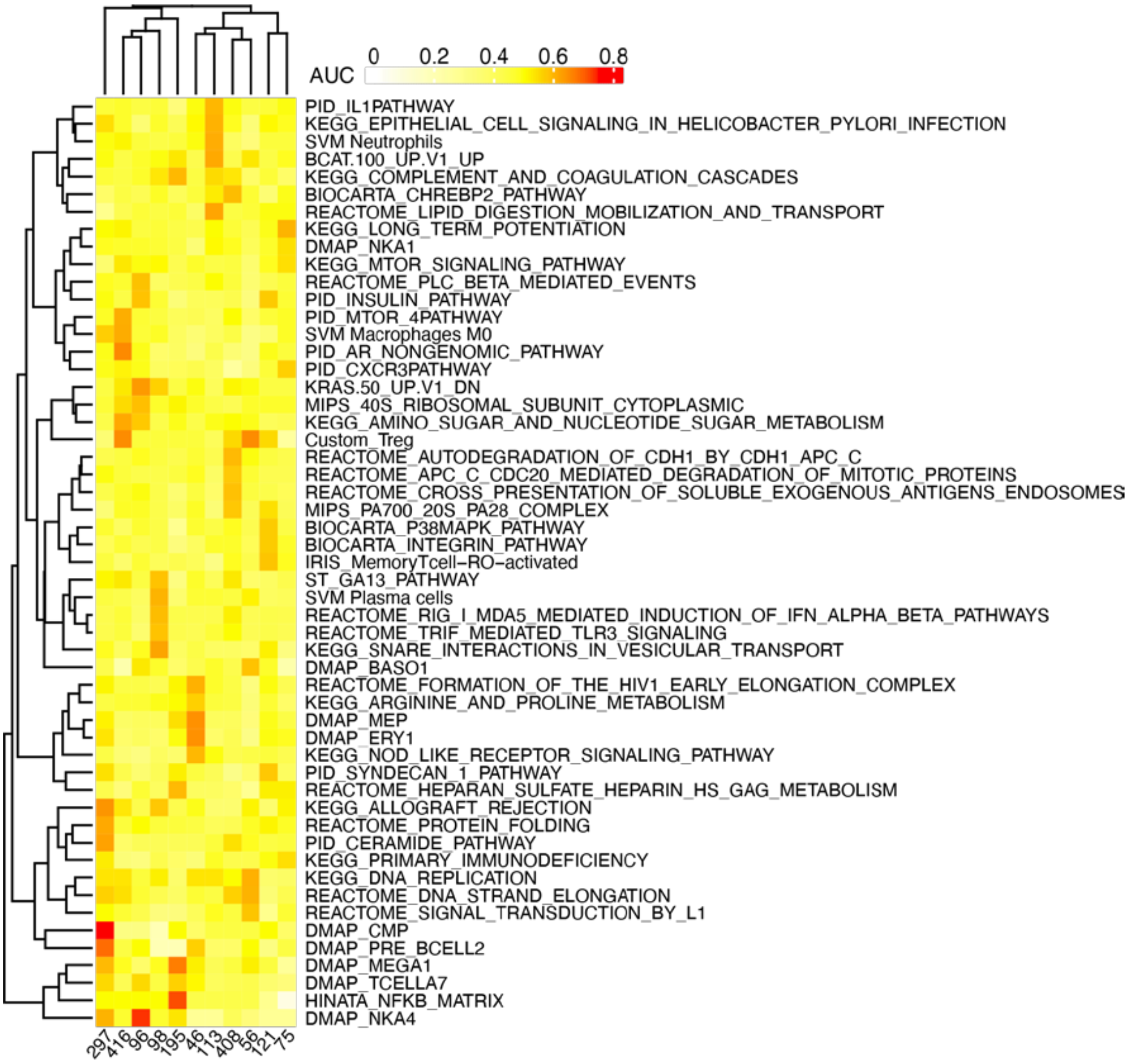

Supplementary Fig. 2. Heatmap of knowledge matrix components of typical latent variables in comparison analysis of GSE39279.

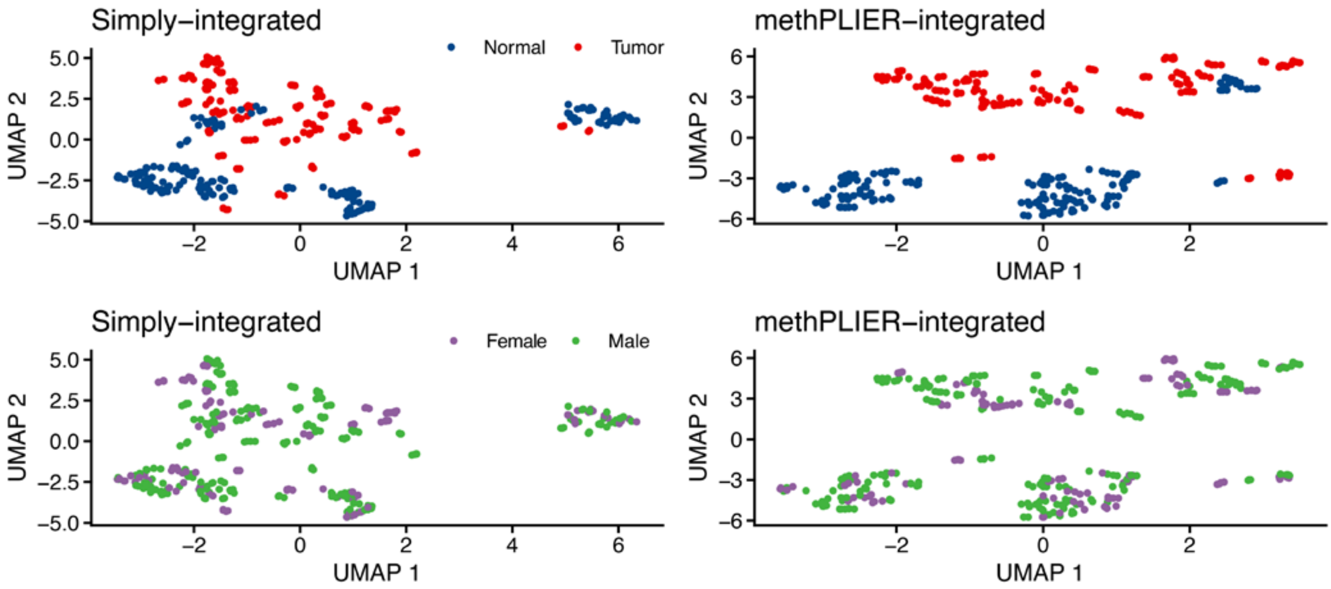

Supplementary Fig. 3. UMAP plot coloring of sample type (cancer/non-cancer) and gender information (female/male) for the dataset in Figure 4B.

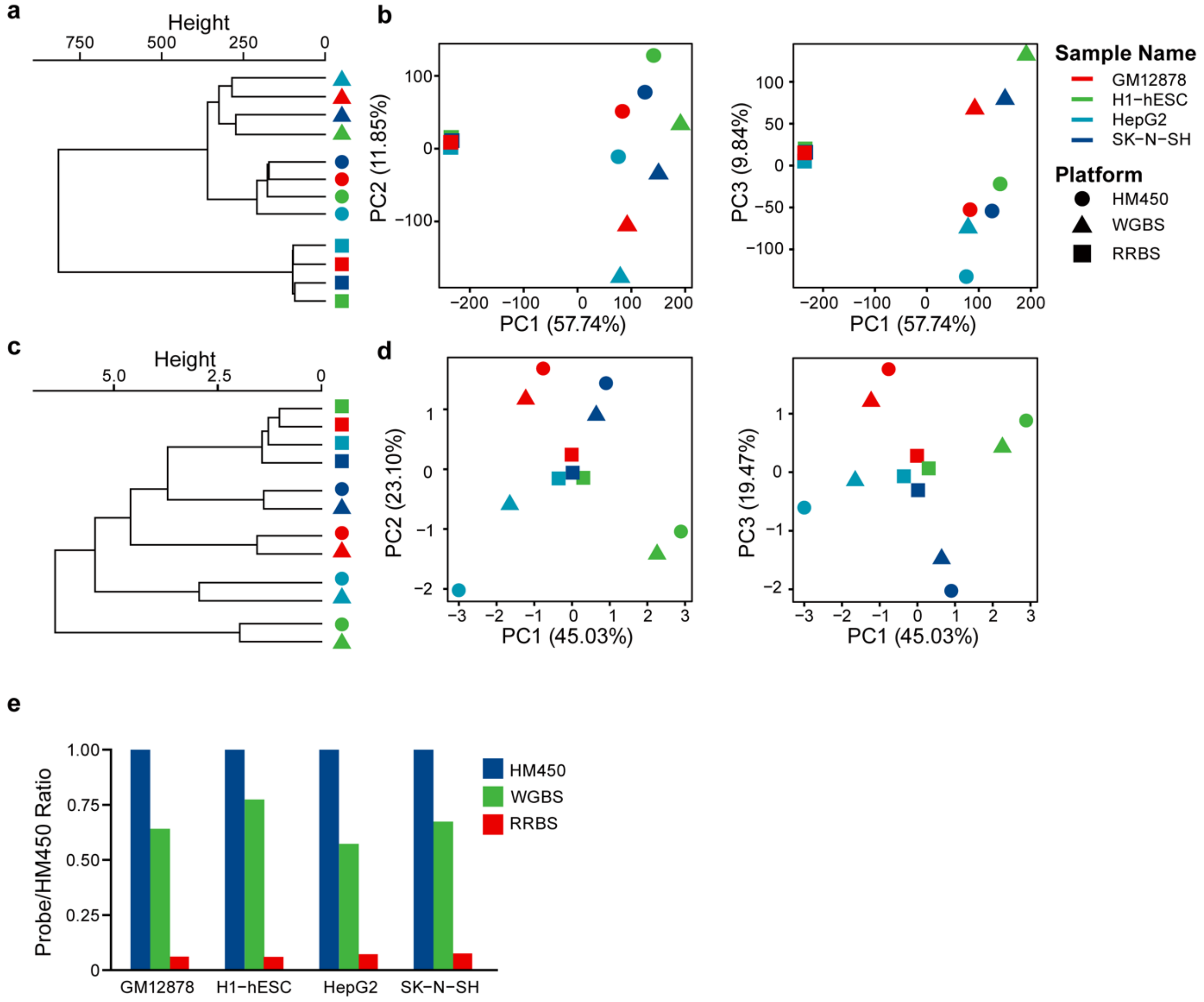

Supplementary Fig. 4. Validation of methPLIER's performance on DNA methylation data acquired from different platforms. HCA and PCA plot of the data integrated without (upper: **a, b**) or with (lower: **c, d**) using methPLIER. The color of the points indicates sample names, where GM12878 is red, H1-hESC is green, HepG2 is light blue, and SK-N-SH is blue. The shape of the points indicates the type of platform used to acquire data, where HM450 is a circle, WGBS is a triangle, and RRBS is a square. (**e**) Bar plot depicting the number of WGBS and RRBS probes corresponding to HM450 probes across various cell lines. The vertical axis indicates the proportion of probes relative to HM450, while the horizontal axis identifies each cell line. Color coding is used to differentiate the data sources, where blue bars represent HM450, green represents WGBS, and red represents RRBS. This visualization provides a clear comparison of probe compatibility with HM450 across different sequencing methods, highlighting the disparities in alignment for each analyzed cell line.

20

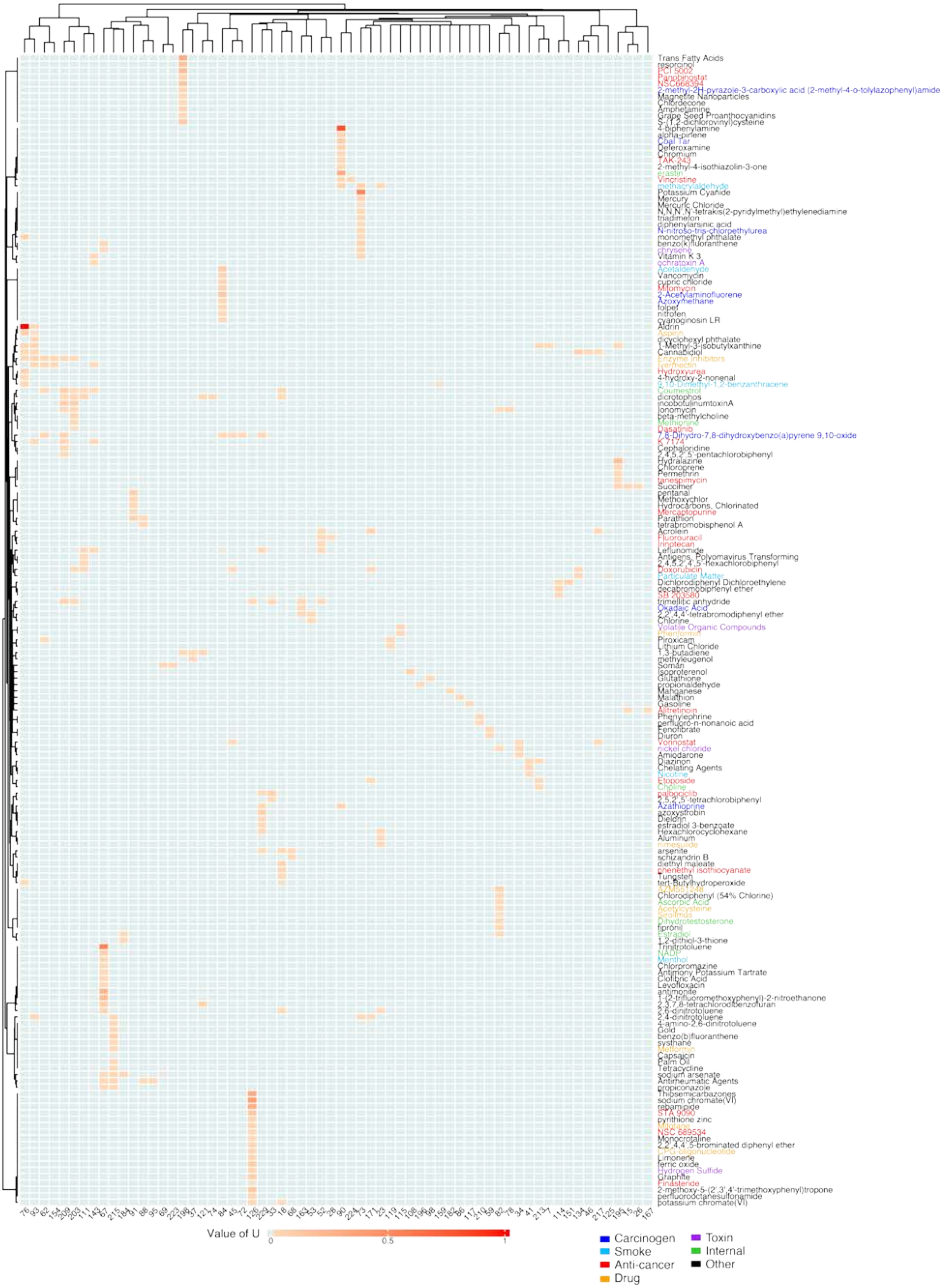
Value of U
0
0.5
1
Carcinogen
Smoke
Anti-cancer
Drug
Toxin
Internal
Other

Supplementary Fig. 5. Heatmap showing factor loadings for each gene set in LV with significant differences between EGFR mutation cases and pan-negative cases in methPLIER-CTD. Each row indicates gene set, each column indicates LV. Larger factor loadings are shown in red, and zero factor loadings are shown in light blue. The following letter colors are assigned according to gene set categories: carcinogen (blue), smoke (light blue), anti-cancer (red), other drugs (orange), toxin (purple), internal small molecule (green), other (black).
